# Bibliometric analysis and visualized study of research on autophagy in ischemic stroke

**DOI:** 10.3389/fphar.2023.1232114

**Published:** 2023-09-04

**Authors:** Jiefang Chen, Gaijie Chen, Xiaojing Xu, Long Chen, Jiewen Zhang, Feng Liu

**Affiliations:** ^1^ Department of Neurology, Henan Provincial People’s Hospital, People’s Hospital of Zhengzhou University, Zhengzhou, China; ^2^ Health Management Center, Henan Provincial People’s Hospital, People’s Hospital of Zhengzhou University, Zhengzhou, China; ^3^ Department of Respiratory and Critical Care Medicine, Henan Provincial People’s Hospital, People’s Hospital of Zhengzhou University, Zhengzhou, China; ^4^ Department of Operating Room, Central China Fuwai Hospital of Zhengzhou University, Zhengzhou, China; ^5^ Department of Nephrology, Henan Provincial People’s Hospital, People’s Hospital of Zhengzhou University, Zhengzhou, China

**Keywords:** bibliometric analysis, visualization analysis, ischemic stroke, autophagy, web of science

## Abstract

**Aims:** To summarize and clarify the current research status and indicate possible future directions in the field of autophagy in ischemic stroke, we performed a comprehensive and multidimensional bibliometric analysis of the literature in this field published from 2011 to 2022.

**Methods:** We retrieved articles on the field of autophagy in ischemic stroke published between 2011 and 2022 from Web of Science Core Collection (WOSCC). VOSviewer (version 1.6.19) and CiteSpace (version 6.2.R2 Basic) were used to identify the leading topics as well as generate visual maps of Countries/regions, organizations, authors, journals, and keyword networks in the related field.

**Results:** A total of 568 publications were contained in this research. The journal with the most publications were Front Pharmacol, Mol Neurobiol, and Neuroscience. China was the most productive country with respect to co-authorship, with the Capital Med Univ being the organization with the most. co-authorships. In terms of authorship analysis, eight of the top 10 most contributive authors were from China. The co-occurring author keywords can be divided into three main clusters, including “protective effect of autophagy in ischemic stroke,” “autophagy-targeted therapy for ischemic stroke,” and “mitochondrial function in cerebral ischemia-reperfusion injury”.

**Conclusion:** This bibliometric analysis helps us reveal the current research hotspots in the research field of autophagy in ischemic stroke and guide future research directions. Subsequent trends in this special field are likely to identify and develop novel autophagy-targeted therapy strategies to effectively prevent and treat ischemic stroke.

## 1 Introduction

Ischemic stroke is considered to be a major cause for the central nervous system dysfunction with high mortality and morbidity rates, which brings heavy burden to public health (GBD 2016 Neurology Collaborators, 2019; Zhou et al., 2019; Koton et al., 2022). Currently, intravenous thrombolysis, arterial thrombolysis, and mechanical thrombectomy are the main effective therapeutic methods for vascular recanalization in ischemic stroke (Wu et al., 2020). Frustratingly, only less than 5% of patients with ischemic stroke can receive timely vascular recanalization treatment due to the narrow treatment time window (within 4.5 h post stroke), among which more than half of them will suffer deterioration or death (Wu et al., 2020; Li et al., 2022). Recent research evidence suggests that insufficient supply of oxygen, glucose, as well as other essential nutrients during ischemia may lead to irreversible brain damage (Campbell et al., 2019). Furthermore, there is also a mechanistic link between brain ischemia and various immune cells, as well as the gut microbiota in altering the brain responses to ischemic insult (Pan et al., 2014; Benakis et al., 2016). Despite many efforts has been made to investigate the molecular pathogenesis of ischemic stroke, there remain unanswered questions (Hankey, 2017; Wang et al., 2018). For example, whether therapeutic transplantation of fecal microbiota normalizes dysbiosis after stroke can improve stroke prognosis (Singh et al., 2016)? Also, whether B lymphocytes play a protective or pathogenic role in cerebral ischemia-reperfusion injury (Liesz et al., 2009; Khoshnam et al., 2017)? Therefore, there is an urgent need to explore novel therapeutic approaches and implementing them into clinical practice to prevent and/or treat ischemic stroke.

Autophagy is considered as a conserved self-cannibalistic catabolic lysosomal degradation pathway that guarantees cellular homeostasis through degrading intracellular long-lived proteins as well as damaged organelles (Klionsky, 2007; Pi et al., 2021). Under nutritional deficiency or metabolic stress, autophagy is activated to maintain normal tissue homeostasis (Klionsky et al., 2021). The breakdown of cellular material by lysosomal enzymes releases amino acids, fatty acids, and other molecules that can be reused by the cell for energy or to build new cellular components (Klionsky, 2007; Klionsky et al., 2021). Maintenance of adequate levels of autophagy are also essential for brain physiopathology (Jimenez-Sanchez et al., 2022). Numerous studies have shown that alteration in autophagy level is regarded as a promising therapeutic strategy for treating various nervous system disease, such as neuroprotection after injury (Sheng et al., 2010; Wang et al., 2012; Puglisi-Allegra et al., 2023), stroke rehabilitation (Han et al., 2021), and ischemic stroke (Wang et al., 2018; Zhu et al., 2022). Although autophagy plays a key role in brain pathophysiology, its role may vary with different nervous system disease. For example, moderate autophagy is considered as an endogenous mechanism for protecting neurons in traumatic brain injury (Erlich et al., 2007), whereas excessive autophagy exacerbates neuronal injury (Lipinski et al., 2015; Wei et al., 2023). Furthermore, another study found that microglia-specific overexpression of PGC-1α could promote poststroke rehabilitation through enhancing autophagy and mitophagy (Han et al., 2021). Furthermore, increasing evidence has also demonstrated that autophagy is activated in various cell types in cerebral tissue during ischemic stroke period (Puyal and Clarke, 2009; Wang et al., 2018; Mo et al., 2020; Peng et al., 2022). Nevertheless, whether activation of autophagy process is a friend or a foe in the pathogenesis of ischemic stroke is still controversial. Specifically, it was previously demonstrated by Zhang et al. that cerebral ischemia-reperfusion-induced autophagy protected against neuronal injury probably through mitochondrial clearance and inhibition of downstream neuronal apoptosis (Zhang et al., 2013). And *in vivo* administration of Mdivi-1, the mitophagy inhibitor, during the reperfusion stage deteriorated the ischemia triggered neuronal damage (Zhang et al., 2013). Whereas, Han et al. revealed that knockdown of circHECTD1 inhibited astrocytes activation in ischemic stroke via targeting MIR142-TIPARP axis through preventing autophagy activation (Han et al., 2018). Hence, target inhibition of circHECTD1 is expected to be a potential therapeutic target for the blocking of astrocyte activation in stroke patients (Han et al., 2018), which still warrants further clinical trials to confirm its efficacy in the future. The above research results indicate that autophagy may play diverse roles in different processes of cerebral ischemia-reperfusion and in different cell types, and it is clear that the research community currently has not yet reached a consensus on the exact role of autophagy in stroke (Wang et al., 2018; Peng et al., 2022).

Despite in-depth research into autophagy and its role in pathophysiological processes, the current awareness of autophagy in ischemic stroke is still in its preliminary stage. Research in the role of autophagy in ischemic stroke is active and promising, therefore a better understanding of autophagy is of great significance to find novel approaches to prevent or treat ischemic stroke. In the past decade or so, we have witnessed a growing number of studies focused on autophagy in ischemic stroke (Ahsan et al., 2021; Hu et al., 2022; Peng et al., 2022; Song et al., 2022; Xiaoqing et al., 2023). Whereas, the explosive growth of publications may prevent researchers from obtaining a large amount of information without fully understanding key developments as well as future directions in the field of autophagy in ischemic stroke. Therefore, a systematic analysis of the hot spots and trends within this special field is warranted. Bibliometrics is a set of methods to quantitatively analyze academic literature in a designated research field employing mathematical and statistical methods (Guler et al., 2016; Chen and Song, 2019). This approach can be used to assess the research trend in a certain special field of study recently as well as predict promising directions for future scientific inquiry in many disciplines (Chen, 2004; Chen et al., 2014; Chen and Song, 2019). Accordingly, bibliometrics analysis has been previously employed to evaluate the development trend and several hotspots of research on the field of ischemic stroke research (Ravindran et al., 2019; Wang et al., 2023; Zhu et al., 2023). However, there is still lacking in-depth bibliometric analysis of autophagy in ischemic stroke area.

Herein, the current study was conducted to systematic analysis of the relevant literature in the field of autophagy in ischemic stroke through utilizing a bibliometric approach to assess the status of current research focus and new research trends, which intended to provide direction for future research.

## 2 Methods

### 2.1 Data collection and search strategies

Data collection were conducted as previously studies described (Agarwal et al., 2016; Chen and Song, 2019). Data for this study were drawn from Web of Science Core Collection (WOSCC) database, which is considered as the most important source of data for bibliometric analysis (Vanzetto and Thomé, 2019; Zhang et al., 2021). Due to there were only a few scattered studies published in the field of autophagy in ischemic stroke before 2011. Thus, we analyzed publications from 2011 to 2022 in the current study. The literatures regarding autophagy in ischemic stroke published between 1 January 2011 and 31 December 2022 were retrieved on 20 March 2023. Primary search terms were “ischemic stroke, cryptogenic ischemic stroke, cryptogenic stroke, cryptogenic embolism stroke, wake up stroke” and “autophagy, macro-autophagy, micro-autophagy, chaperone-mediated autophagy.” Only “research articles” and “review articles” published in English were considered. In order to acquire studies related to autophagy in ischemic stroke in the past 12 years, we performed the following search strategies: #1 (TS = (“acute ischemic stroke” OR “ischemic stroke” OR “cryptogenic ischemic stroke” OR “cryptogenic stroke” OR “cryptogenic embolism stroke” OR “wake up stroke” OR “wake-up stroke”)); #2 (TS = (“autophagy” OR “macroautophagy” OR “microautophagy” OR “chaperone-mediated autophagy” OR “autophagocytosis” OR “reticulophagy” OR “ER-Phagy” OR “nucleophagy” OR “ribophagy” OR “lipophagy” OR “mitophagy”)); #3 (#1 and #2). In total, 568 papers regarding autophagy in ischemic stroke were identified in the WOSCC between 2011 and 2022, mainly including 436 original research articles and 132 reviews.

### 2.2 Analysis and visualization

Bibliometric analysis and data visualization were mainly performed with VOSviewer software (version 1.6.19) and CiteSpace software (version 6.2.R2 Basic). Using VOSviewer software, we systematically generated knowledge maps of the citation networks among countries/regions, various organizations, authors, co-citation authors, as well as keywords co-occurrence networks (van Eck and Waltman, 2010; Zhao et al., 2022; Zhou et al., 2022). Whereas, CiteSpace primarily uses time slicing technology to establish a time series of network models that changes over time and combines these individual networks to form an overview network for systematic study of relevant literature. Through using CiteSpace software, we have got visualization knowledge maps of cooperation among institutes, dual-map overlay of journals, cluster view of co-cited references, as well as top references with the strongest citation bursts and top keywords with the strong citation bursts (Zhu et al., 2020; Liu et al., 2022; Pei et al., 2022). Additionally, keywords were clustered in every period to determine the research emphasis and relevant changes. And “burst detection” refers to functions provided by the CiteSpace software to identify emerging trends and sudden changes in a specific field (Assenov et al., 2008). In addition, in order to administrate the raw data downloaded from WoSCC database, the software Excel Microsoft Office 2020 was also employed. Furthermore, our data collection of articles in the field of autophagy in ischemic stroke meets the minimum sample size requirement (no less than 200 papers) for bibliometric analysis as previous study recommended (Rogers et al., 2020).

## 3 Results

### 3.1 The annual trend of paper publication quantity and citations

**FIGURE 1:**
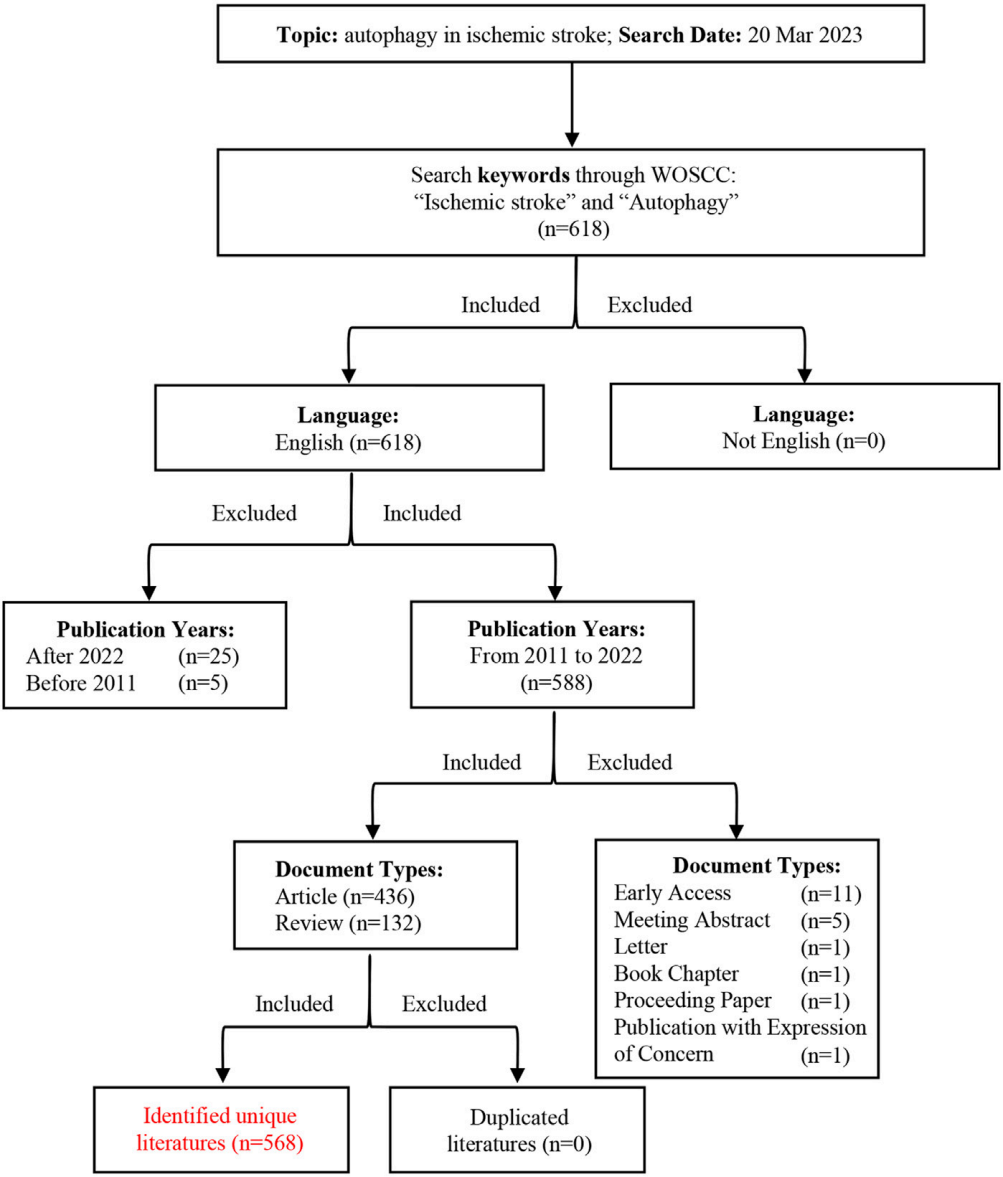
Flow chart displaying the process of study identification.

**FIGURE 2:**
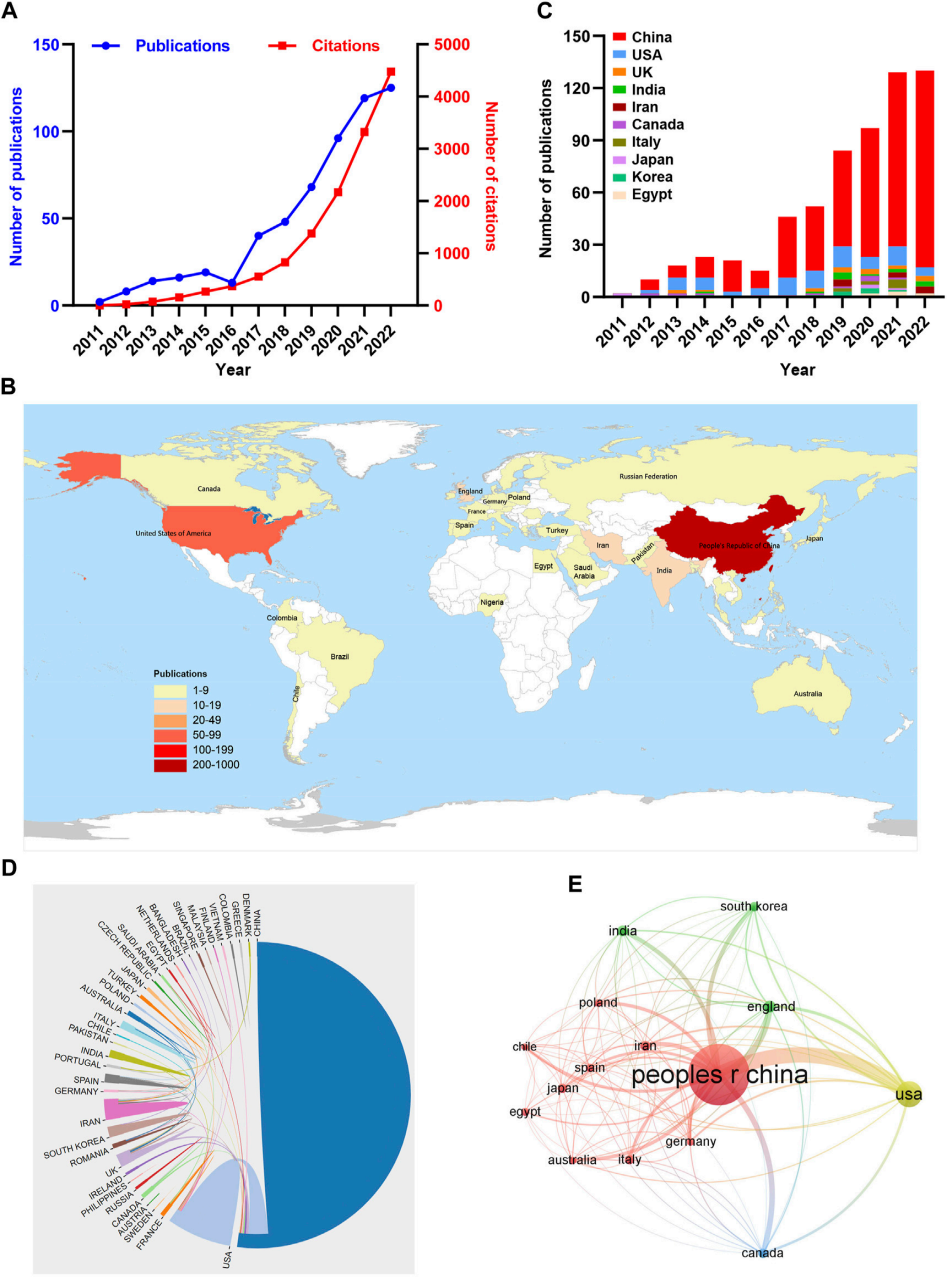
Cooperation among countries/regions that published autophagy in ischemic stroke-related studies from 2011 to 2022. **(A)** The annual number and total citations of articles related to autophagy in ischemic stroke from 2011 to 2022. **(B)** Geographical distribution of global output in the field of autophagy in ischemic stroke. **(C)** Annual output trend of the top 10 productive countries in the field of autophagy in ischemic stroke. **(D)** The collaborations among different countries/regions. The links denotes the frequency of the collaborations between countries/regions in the field of autophagy in ischemic stroke. **(E)** VOSviewer software was used to conduct the citation network among different countries/regions in the field of autophagy in ischemic stroke. The size of nodes represents the number of publications and the thickness of links represents the citation strength.

In total, 568 documents regarding autophagy in ischemic stroke research were identified in the WOSCC between 2011 and 2022, mainly including 436 original research articles and 132 reviews. The detailed screening processes were illustrated in Figure 1. To further investigate the growth of this field in the past decade or so, we counted the number of publications every year during this research period. Overall, the number of publications increased steadily in this field between 2011 and 2022 except for 2016, especially after 2017, the number of published papers increased rapidly. In addition, it came to our attention that the number of related publications is stable at more than 100 in 2021 and 2022 (Figure 2A), indicating that this research field has attracted more and more scholars’ attention in recent years. Moreover, the above publications regarding autophagy in ischemic stroke were cited a total of 13,610 times, with an average of 23.96 citations per paper and an h-index of 59 (20 March 2023).

### 3.2 Countries/regions and organizations analysis

As shown in world map of Figures 2A,B total of 40 countries/regions have published related articles in the field of autophagy in ischemic stroke during the past 12 years. The top 10 productive countries/regions in the research field of autophagy in ischemic stroke are illustrated in Table 1 and Figure 2C, with China (470), the United States (78) and United Kingdom (16) contributing the most. However, citation analysis indicated that Canada has the highest average citation per article (58.00), followed by United States (42.17) and United Kingdom (38.56) (Table 1). When it comes to collaborations among different countries/regions analysis, there were a lot of cooperations among different countries/regions, especially China and the United States, indicating that they played a crucial role in the cooperation between countries, which may be related to the different culture and academic atmosphere in different countries/regions (Figure 2D).

**TABLE 1:** Top 10 productive countries in the research field of autophagy in ischemic stroke.

Rank	Country	Publications (%)	Average citation	H-index
1	China	470 (66.48%)	23.85	54
2	United States	78 (11.03%)	42.17	36
3	United Kingdom	16 (2.26%)	38.56	10
4	India	12 (1.70%)	20.17	7
5	Iran	11 (1.56%)	23.64	5
6	Canada	9 (1.27%)	58.00	9
7	Italy	9 (1.27%)	32.56	7
8	Japan	9 (1.27%)	28.11	7
9	Korea	9 (1.27%)	29.00	6
10	Egypt	7 (1.01%)	6.29	4

Citation analysis, as a quantitative bibliometric method, can be used to comprehensive evaluate the influence as well as the importance of an article in a particular field through analyzing the citation characteristics (Agarwal et al., 2016). In the VOSviewer citation network map, different color nodes indicate different countries/regions and a larger node represents more publications in the country/region. The links represent the citation strength between different countries/regions on autophagy in ischemic stroke research. The citation network map in Figure 2E displayed the citation relationships among the top 15 countries/regions which published at least five documents. Based on citation networks, we identified that China, United States, United Kingdom, Canada, Iran, and Germany had the most frequent citation relationship, indicating that articles published by these countries possess a certain level of influence in the field.

**FIGURE 3:**
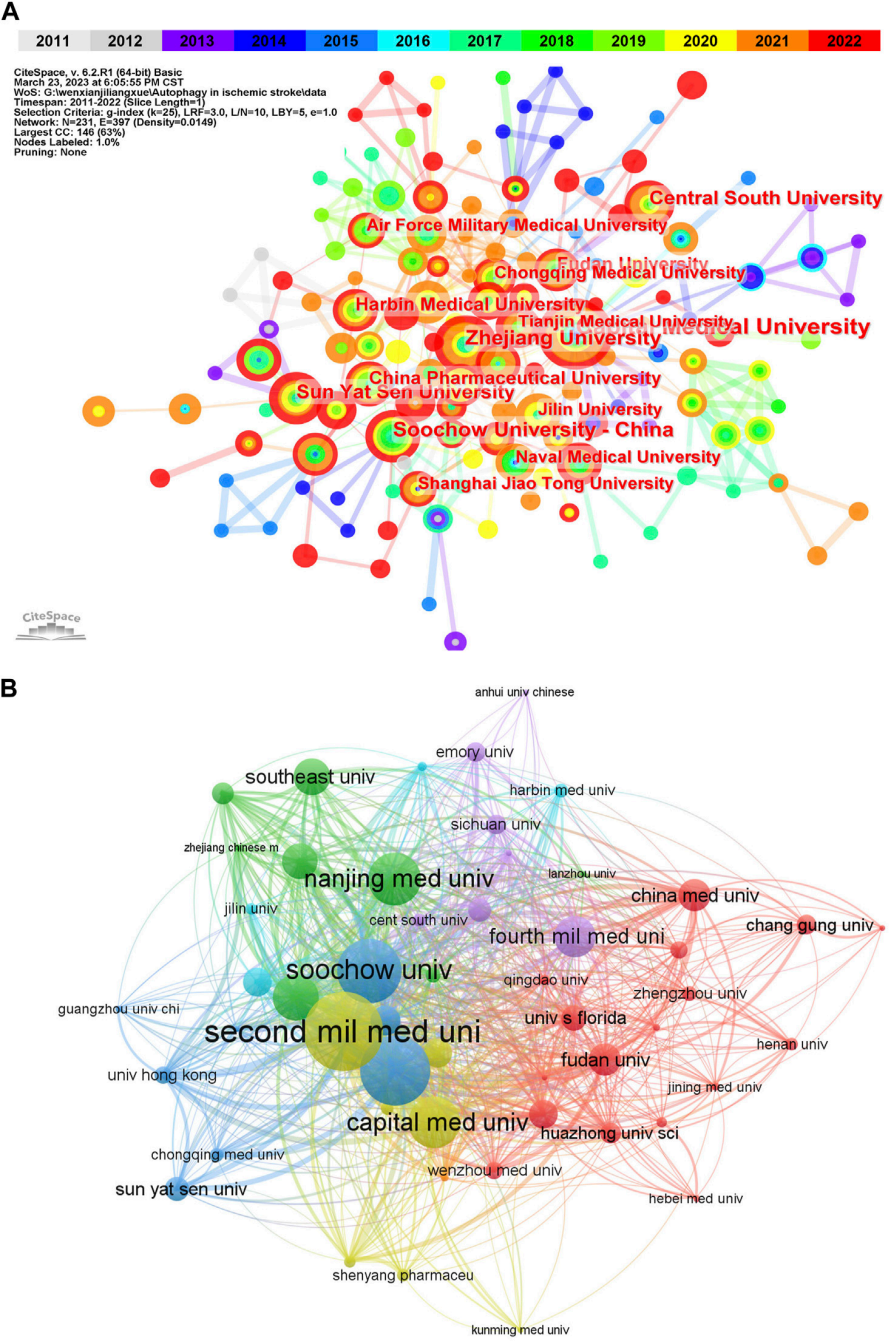
Visualization of active institutes in the field of autophagy in ischemic stroke. **(A)** Cluster analysis of cooperation among institutes generated by CiteSpace. N = 231, E = 397 (N represents the number of network nodes and E represents the number of connections). **(B)** The citation network of institutions generated by VOSviewer.

According to VOSviewer analysis, the above 568 publications were contributed by 690 different organizations, among which the top 10 institutions with the published papers were listed in Table 2. And it could be found that China occupied all of the top 10 institutions, which may be contributed to the close collaboration among these organizations (Figure 3A). These findings suggest that China has occupied a core position in this field of research in the whole network. It is also reasonable to expect that strengthening cooperation with Chinese institutions can promote the progress of research in this field. Besides, from the co-occurrence map of institutions, we can also discover that the connections of years colored by yellow and red were the most widely distributed, indicating that 2020 and after were the most intensive years of inter-institution cooperation. When it comes to citation analysis of organizations, the Second Mil Med Univ had the largest number of citations (915), followed by and Shanghai Jiao Tong Univ (806) Soochow Univ (748) (Figure 3B).

**TABLE 2:** Top 10 productive organizations in the research field of autophagy in ischemic stroke.

Rank	Organization	Publications	Citations	Country/region
1	Capital Med Univ	28	594	China
2	Zhejiang Univ	21	530	China
3	Soochow Univ	19	748	China
4	Sun Yat-sen Univ	16	280	China
5	China Med Univ	14	378	China
6	Fudan Univ	14	373	China
7	China Pharmaceut Univ	13	347	China
8	Harbin Med Univ	13	164	China
9	Tianjin Med Univ	13	270	China
10	Second Mil Med Univ	12	915	China

### 3.3 Contributions of authors and co-cited authors

**FIGURE 4:**
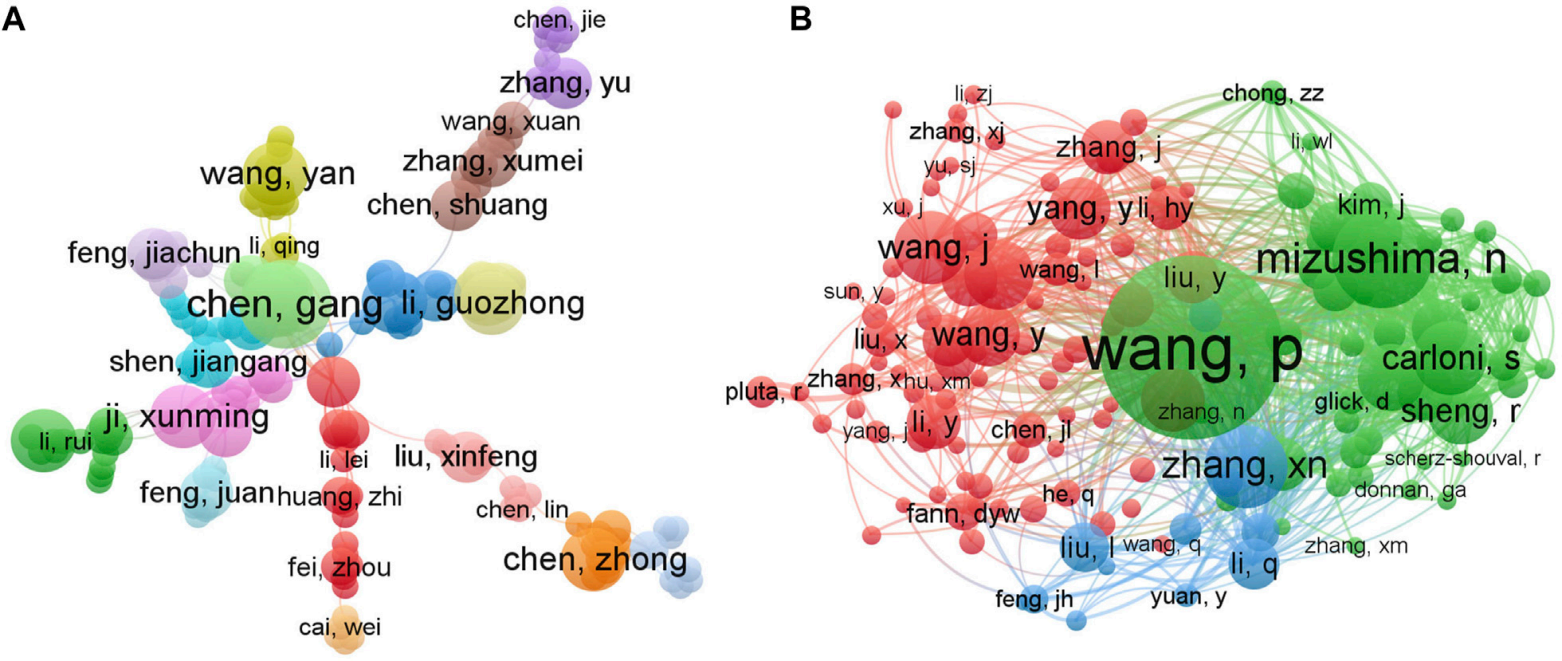
VOSviewer visualization network maps of authors and co-cited authors contributed to the research field of autophagy in ischemic stroke. **(A)** The cooperation network map of authors. Of the 3,438 authors, 116 had at least 3 publications in this field. **(B)** Network map of co-citation authors. Among the 19,810 co-cited authors, 142 had at least 20 citations.

In order to generate a visualization network regarding the authors and co-cited authors of the research of autophagy in ischemic stroke, the VOSviewer visualization software was employed. As illustrated in Figure 4A, a total of 3,438 authors were obtained in this field and different colors represented different clusters. Among those authors, Chen Gang from Soochow University, as the most productive author, published 7 documents, followed by Chen Zhong, Fang Marong, Han Bing, Han Song, Hu Zhiping, Ji Xunming, Miao Chao-yu (5 papers), and Hadley Gina, Bae Ok-Nam (4 papers) (Table 3). However, to our surprise, there was no strong links among these core authors, implying the urgent need for enhanced collaboration and communication to further drive rapid developments in the field. In addition, it is worth noting that Miao Chao-yu from the Second Military Medical University in China had the highest number of total citations (Table 3), indicating that his/her research achievements in this field have been widely recognized around the world.

**TABLE 3:** Top 10 productive authors in the research field of autophagy in ischemic stroke.

Rank	Authors	Organizations	Papers	Citations	Countries
1	Chen Gang	Soochow Univ	7	256	China
2	Chen Zhong	Zhejiang Univ	5	198	China
3	Fang Marong	Zhejiang Univ	5	82	China
4	Han Bing	Southeast Univ	5	401	China
5	Han Song	Capital Med Univ	5	270	China
6	Hu Zhiping	Cent South Univ	5	83	China
7	Ji Xunming	Capital Med Univ	5	140	China
8	Miao Chao-yu	Second Mil Med Univ	5	660	China
9	Hadley Gina	Univ Oxford	4	210	United Kingdom
10	Bae Ok-Nam	Hanyang Univ	4	221	Korea

The co-citation relationship network of authors with at least 20 citations mainly displayed three clusters, showing the authors with a significant influence in the field of autophagy in ischemic stroke research (Figure 4B). Based on the co-citation analysis, we revealed that the top three most cited authors were Wang Pei (215 times, from Second Military Medical University), Noboru Mizushima (118 times, from Tokyo Medical and Dental University), and Zhang xiangnan (98 times, from Zhejiang University). Moreover, the top three authors in terms of the total link strength (TLS) were also Wang Pei (TLS = 3053), Noboru Mizushima (TLS = 2087), and Zhang xiangnan (TLS = 1621), indicating that these three scholars hold an authoritative position in the research field of autophagy in ischemic stroke, which can provide valuable reference for later researchers in this field.

### 3.4 Journal analysis

A total of 207 journals covered papers on this topic, including 38 journals that published at least 5 documents. The top ten journals were illustrated in Table 4, which covered 113 articles in the field of autophagy in ischemic stroke, accounting for 20.0% of the total publications. Among the top 10 journals, 4 journals were at the Q1 JCR division according to the 2022 edition of the Journal Citation Reports (JCR), including Frontiers in Pharmacology (impact factor = 5.99), Molecular Neurobiology (impact factor = 5.69), CNS Neuroscience & Therapeutics (impact factor = 7.04), and Biomedicine & Pharmacotherapy (impact factor = 7.42). Frontiers in Pharmacology (14 papers), Molecular Neurobiology (14 papers), and Neuroscience (14 papers) contributed the most articles, while Circulation was the journal with highest impact factor in 2022 (39.92).

**TABLE 4:** Top 10 productive journals in the field of autophagy in ischemic stroke.

Rank	Journal	Publications	Citations	IF (2022)	JCR
1	Front Pharmacol	14	76	5.99	Q1
2	Mol Neurobiol	14	278	5.69	Q1
3	Neuroscience	14	494	3.71	Q3
4	CNS Neurosci Ther	13	532	7.04	Q1
5	Int J Mol Sci	11	200	6.21	Q2
6	Brain Res	10	188	3.61	Q3
7	J Ethnopharmacol	10	127	5.2	Q2
8	Biochem Bioph Res Co	9	478	3.32	Q3
9	Biomed Pharmacother	9	264	7.42	Q1
10	Front Neurol	9	50	4.09	Q2

**FIGURE 5:**
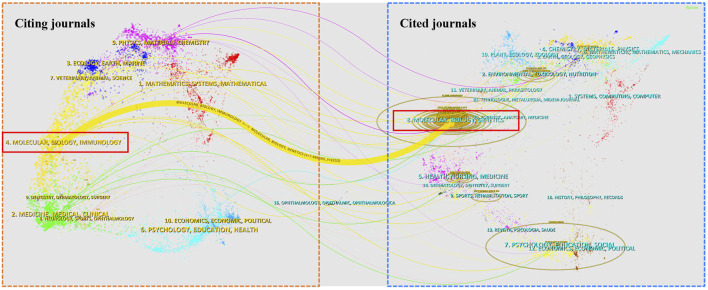
Journal analysis in the research field of autophagy in ischemic stroke. The dual-map overlay of journals on autophagy in ischemic stroke generated by CiteSpace software. Specifically, the labels represented different research subjects covered by the journals. Different colored lines correspond to the different paths of references, starting from the citing journals (left half) to the cited journals (right half). The main citing journals were shown in the red box and the main cited journals were shown in the blue box.

As illustrated in Figure 5, the dual-map overlay of the citing journals and cited journals related to autophagy in ischemic stroke researches indicated the subject distribution of these journals, with the left half of the graph symbolizing citing journals and the other half representing cited journals. Furthermore, the colored lines indicated the citation relationship between articles in citing journals and articles in cited journals. From the result, it could be seen that one of the main citation paths was from “Molecular, Biology, Genetics” (co-cited journals) to “Molecular, Biology, Immunology” (citing journals), indicating that autophagy in ischemic stroke-related researches is mainly focused on the field of basic research, with a special focus on genetics and immunity, but research on clinical transformation is still very limited.

### 3.5 Co-cited references analysis

In general, 28,477 references were cited by researches published on the field of autophagy in ischemic stroke, among which the top 10 highly cited references were listed in Table 5 (Carloni et al., 2008; Wen et al., 2008; Puyal et al., 2009; Sheng et al., 2010; Kim et al., 2011; Shi et al., 2012; Wang et al., 2012; Wei et al., 2012; Zhang et al., 2013; Wang et al., 2018). The research entitled “Cerebral ischemia-reperfusion-induced autophagy protects against neuronal injury by mitochondrial clearance” published in Autophagy in 2013 ranked first with 73 co-citations (Zhang et al., 2013), followed by the researches “Autophagy in ischemic stroke” published in Progress In Neurobiology in 2018 (71 co-citations) (Wang et al., 2018) and “Neuronal injury in rat model of permanent focal cerebral ischemia is associated with activation of autophagic and lysosomal pathways” published on the journal Autophagy in 2013 with 67 co-citations (Wen et al., 2008). Among the top 10 co-cited articles, four of which were published in well-known and high reputable journals in the field of autophagy, Autophagy (Wen et al., 2008; Sheng et al., 2010; Wang et al., 2012; Zhang et al., 2013), which is dedicated to the study of the process of autophagy and focuses on cutting-edge research on this rapidly evolving field.

**TABLE 5:** Top 10 highly cited publications in the field of autophagy in ischemic stroke.

Rank	Tittle of reference	Journal	Citation	Year	IF
1	Cerebral ischemia-reperfusion-induced autophagy protects against neuronal injury by mitochondrial clearance	Autophagy	73	2013	13.39
2	Autophagy in ischemic stroke	Prog Neurobiol	71	2018	10.89
3	Neuronal injury in rat model of permanent focal cerebral ischemia is associated with activation of autophagic and lysosomal pathways	Autophagy	67	2008	13.39
4	Induction of autophagy contributes to the neuroprotection of nicotinamide phosphoribosyl transferase in cerebral ischemia	Autophagy	54	2012	13.39
5	Excessive autophagy contributes to neuron death in cerebral ischemia	CNS Neurosci Ther	45	2012	7.04
6	Protective role of autophagy in neonatal hypoxia-ischemia induced brain injury	Neurobiol Dis	44	2008	7.05
7	Autophagy activation is associated with neuroprotection in a rat model of focal cerebral ischemic preconditioning	Autophagy	43	2010	13.39
8	Postischemic treatment of neonatal cerebral ischemia should target autophagy	Ann Neurol	42	2009	11.27
9	AMPK and mTOR regulate autophagy through direct phosphorylation of Ulk1	Nat Cell Biol	38	2011	28.21
10	A double-edged sword with therapeutic potential: an updated role of autophagy in ischemic cerebral injury	CNS Neurosci Ther	38	2012	7.04

**FIGURE 6:**
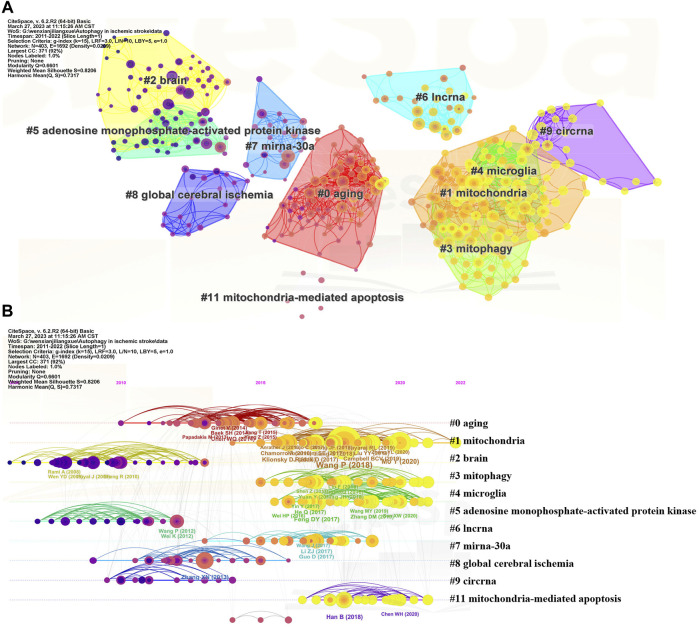
Visualization of co-cited reference analysis regarding autophagy in ischemic stroke researches. **(A)** Cluster view of co-cited references using keywords as label source. **(B)** Timeline distribution of the top 10 clusters.

To further explore hot topics in autophagy in ischemic stroke-related research, the co-citation network maps of references were drawn using CiteSpace software and main clusters with keywords were identified (Figures 6A,B). As illustrated in Figure 6A, the 10 main clusters were symbolized with different background colors and node types, which represented the number and centrality of the co-cited references. Similarly, in Figure 6B, node position represented the time of scientific references and node size denoted the reference total citations. As can be seen from the timeline view, “mitochondria (#1),” “mitophagy (#3),” “microglia (#4),” and “mitochondria-mediated apoptosis (#11)” were recent clusters, indicating the current research hotspots of autophagy in ischemic stroke area.

**FIGURE 7:**
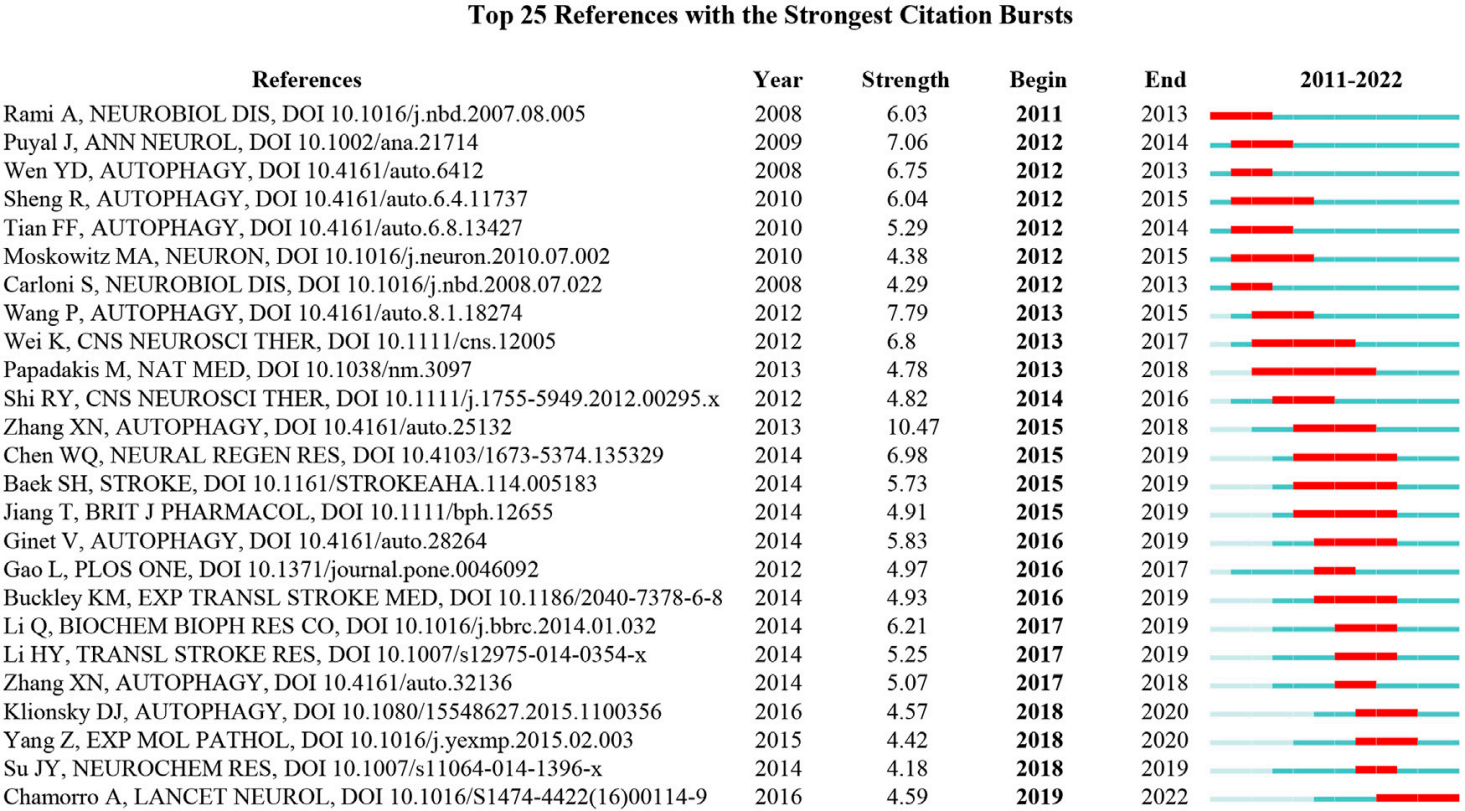
The top 25 references with the strongest citation bursts during 2011–2022.

Major milestone in the development of autophagy in ischemic stroke area can be recognized from the list of top 25 references that possess strong citation bursts between 2011 and 2022 (Figure 7). As suggested by professor Chaomei Chen, references presented strong values in the strength column tend to be a landmark milestone for the science mapping research (Chen, 2004). Among these citations, the earliest reference with the strongest citation bursts was “Rami A, 2008, NEUROBIOL DIS, DOI 10.1016/j.nbd.2007.08.005” (Rami et al., 2008), with citation bursts from 2011 to 2013. The reference “Chamorro A, 2016, LANCET NEUROL, DOI 10.1016/S1474-4422 (1600114-9”) maintained citation peaks until 2022 (Chamorro et al., 2016), which was related to neuroprotection in acute stroke, indicating that this field is still a hot research spot for current.

### 3.6 Analysis of keywords

**FIGURE 8:**
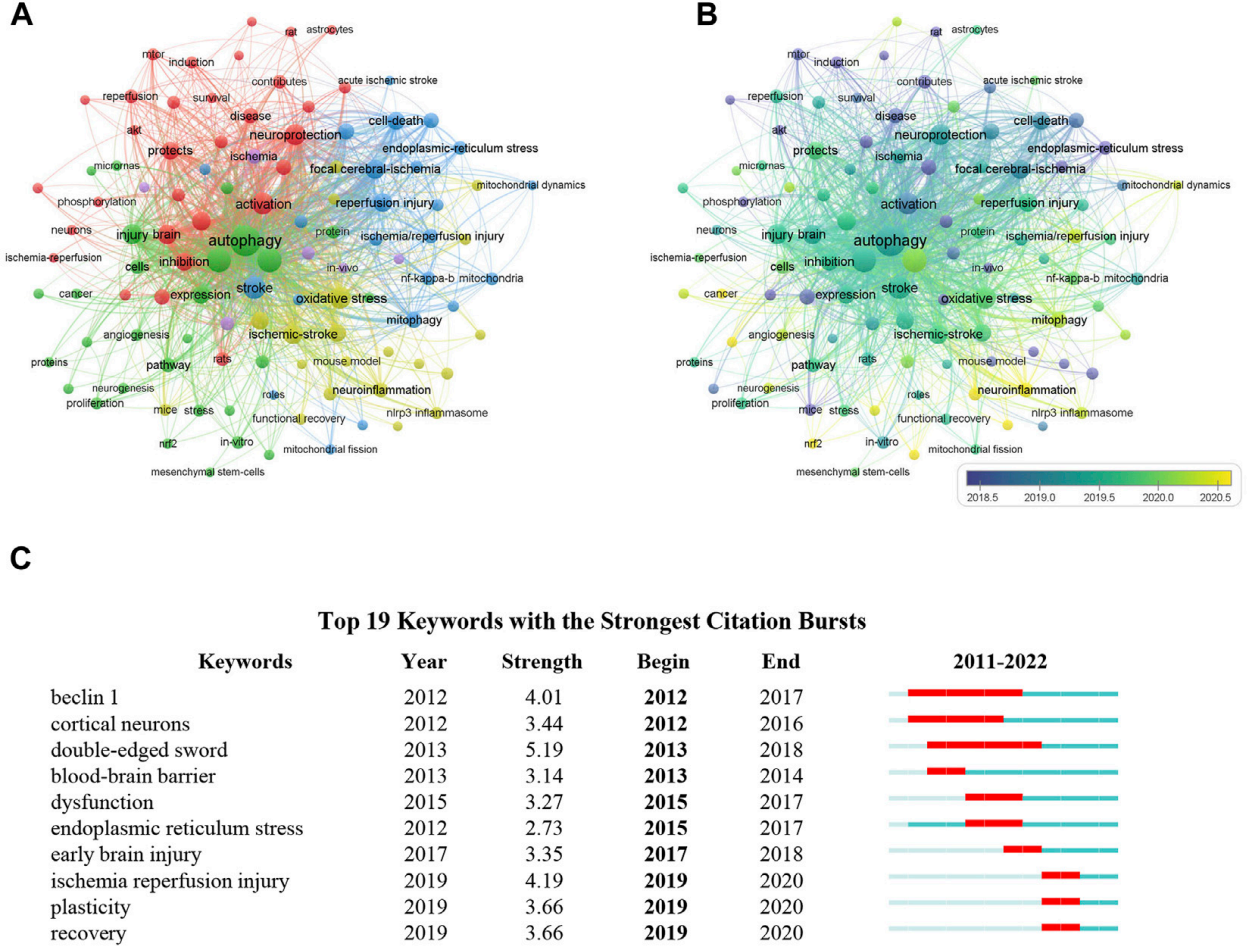
Visualization of keyword analysis in the field of autophagy in ischemic stroke. **(A)** The co-occurrence network map of keywords. Those keywords with strong correlation were grouped into 5 clusters as indicated by different colors. **(B)** The overlay visualization map showed that keywords were colored according to the mean year the keywords appeared in the articles. **(C)** Top 10 keywords with the strong citation bursts from 2011 to 2022.

Given that the keywords in a scientific article often reflect the topic of research, it is likely to distinguish important topics as well as the emerging trends in a specific research area through analyzing the frequency and co-occurrence of keywords (van Eck and Waltman, 2010; Dos Santos et al., 2022). Thus, VOSviewer software was employed to determine the co-occurrence of keywords in the field of autophagy in ischemic stroke. In total, 2,557 keywords were identified in documents related to this topic published between 2011 and 2022, of which 108 keywords emerged over 10 occurrences. The most frequent keywords were “autophagy” (375 times), “apoptosis” (180 times), “ischemic stroke” (179 times), “oxidative stress” (119 times, and “neuroprotection” (111 times). For the construction of the co-occurrence network map of the most frequent keywords in publications related to autophagy in ischemic stroke, keywords with at least 10 occurrences were chose. According to Figure 8A, keywords with strong correlation were grouped into 5 clusters as denoted by various colors, of which 3 main groups were identified based on the number of keywords with more than 20. Among these clusters, cluster 1 (in red color) is the largest group and has 31 keywords, which is mainly focused on the protective effect of autophagy on neuronal injury during cerebral ischemia, adding the terms “neuroprotection,” “neuronal autophagy,” “protects,” etc. The cluster 2 (in green color) is mainly related to explore autophagy-targeted therapeutic methods for cerebral ischemia, which primarily includes the keywords “angiogenesis,” “neurogenesis,” “therapy.” The cluster 3 (in blue color) is mainly involved in mitochondrial function and cerebral ischemia-reperfusion injury, adding the terms “mitophagy,” “mitochondrial dysfunction,” “neuronal injury.” In Figure 8B, the keywords overlay map was generated based on the mean year the keywords appeared in the articles. In recent years, “neurogenesis,” “neuroinflammation,” “mitochondrial dynamics,” “mitophagy,” and “neuronal autophagy” appeared frequently, indicating that neuronal mitophagy in cerebral ischemia is a current research focus. Furthermore, the keywords with a strong citation burst are another important signal to indicate the emerging trend of a specific research field. As we can see from Figure 8C, the citation bursts keywords such as double-edged sword (5.19, 2013-2018), endoplasmic reticulum stress (2.73, 2015-2017), and plasticity (3.66, 2019-2020), indicating that these fields may no longer be the current research focus.

## 4 Discussion

In this study, we have performed a systematic and comprehensive bibliometric analysis of studies with respect to autophagy in ischemic stroke field from 1 January 2011 to 31 December 2022. Aa far as we concerned, this is the first in-depth bibliometric study conducted on autophagy in ischemic stroke research and has made certain contributions to this area. Specifically, this study forms a contribution to autophagy in ischemic stroke area that can guide researchers to identify the status of recent research, current research focus, as well as new research trends in this emerging field. Our overall findings for the research of autophagy in ischemic stroke showed that annual publishing output displayed a continuous upward trend between 2011 and 2022, except for a brief setback in 2016 and then experienced a sudden rise in 2017. These results indicate that there is an overall trend of more research findings published over the past 12 years, indicating that increasing researchers have started to focus on the field of autophagy in ischemic stroke research. And this suggests that it will be a novel research hotspot in the foreseeable future and will continue to receive attention.

Although publications are scattered around the world, research from China and the United States predominated in terms of quantity, accounting for 77.5% of all publications, which may be related to the pioneering researchers and significant financial support from China and the United States in this field. While in terms of institutions analysis, China occupied all of the top 10 institutions by the number of publications, which may be explained by the close collaboration among authors in these different institutions in China. Overall, China led the world in the field of autophagy in ischemic stroke, both in the total number of publications on this field and influential research organizations. When it comes to journal analysis, Frontiers in Pharmacology, Molecular Neurobiology, and CNS Neuroscience & Therapeutics ranked among the leading contributors to this field, all of which belong to JCR subregion Q1 with impact factor exceeding 5. Besides, some authoritative journals in the field of neuroscience, such as Translational Stroke Research, Stroke, and Current Neuropharmacology, have also published quite a few high-quality research findings in this field, suggesting the particular important role of autophagy in the ischemic stroke research. The above journal analysis can provide adequate guidance for researchers who submit articles in this field for publication. Moreover, from the comprehensive results of publication outputs and citations, the three most cited journal are Stroke, Autophagy, and Progress in Neurobiology, all of which are the authoritative journals in the related fields, indicating that these studies have sufficient theoretical support. In addition, it is worth noting that the journal Autophagy published fewer documents but contributed the most significant credited citation counts, making it the most influential journal in the field of autophagy-related research. Therefore, breakthrough research in this field can be prioritized for publication in Autophagy, making it easier to gain widespread recognition.

Due to the fact that references are the knowledge base for research, co-citation analysis of the references can facilitate the identification of the knowledge base of the indicated research fields (Zou et al., 2018; Zhao et al., 2022). Among those citations, the most cited one is an article published in Autophagy on 12 June 2013 by Xiangnan Zhang et al. from Zhejiang University, titled “Cerebral ischemia-reperfusion-induced autophagy protects against neuronal injury by mitochondrial clearance”, which has systematically investigated the accurate role of autophagy in the reperfusion stage of ischemic stroke for the first time (Zhang et al., 2013). The results of their research indicate that cerebral ischemia-reperfusion-induced autophagy protected against neuronal injury probably through mitochondrial clearance and inhibition of downstream neuronal apoptosis (Zhang et al., 2013). The second most cited is a review entitled “Autophagy in ischemic stroke” published in Progress in Neurobiology 5 years ago by Pei Wang from Second Military Medical University, which conducted a comprehensive review on the autophagy regulation of neurons, glial cells and cerebral microvascular cells in response to ischemic stress, highlighting that the precise role of autophagy in ischemic stroke is decided by the status of blood flow reperfusion into cerebral during ischemic insult (Wang et al., 2018). These two articles have been extensively cited, presumably due to the primary background knowledge of autophagy in ischemic stroke research is linked to the issues studied in these two articles. In addition, it is worth noting that among the top 10 co-cited articles in the field of autophagy in ischemic stroke, 7 articles were contributed by Chinese scholars, further indicating that strengthening cooperation with those well-known Chinese scholars will greatly promote the development of this field. It is worth noting that, despite the existence of international cooperation, research in this field is mainly concentrated in China and some developed countries, and comprehensive international cooperation is indispensable for promoting the development of this field.

As keywords in a scientific article reflect the general topic of the research, thus important themes and frontier directions in a particular area of research can be identified through dissecting the frequency and co-occurrence of keywords (Dos Santos et al., 2022). Based on the results of keywords clustering analysis, the following suggestions are put forward for future research regarding autophagy in ischemic stroke research field. During ischemic stroke, almost all types of brain cells are involved. The concept of the “neurovascular unit” has provided a basic framework for better understanding the pathology of CNS diseases, including stroke (Arai et al., 2009). Accumulating evidence has indicated that autophagy is closely involved in the pathogenesis and progression of ischemic stroke as a double-edged sword (Moskowitz et al., 2010; Wang et al., 2018). Thus, it is of great clinical significance to investigate the potential role of autophagy in neurovascular unit, such as neurons, astrocytes, microglial cells, to regulate the neurological homeostasis in ischemic stroke. According to the cluster analysis of keywords co-appearance, we found that intracellular biological processes, such as mitochondrial dysfunction, endoplasmic reticulum (ER) stress, and oxidative stress, are hotspots in the field of autophagy in ischemic stroke, all of which have complex associations with autophagy in regulating neuronal death or survival. As a specific type of autophagy, mitochondrial autophagy (mitophagy) is especially crucial in maintaining mitochondrial homeostasis (Galluzzi et al., 2018). Much evidence from recent researches supports that inducing mitophagy can prevent ischemic brain injury (Tang et al., 2016; Ham and Raju, 2017; Han et al., 2021), so developing targeted mitophagy methods can serve as a potential strategy for treating ischemic stroke. Whereas, how can we accurately real-time monitor mitophagy during ischemic stroke process remain to be solved urgently. Moreover, although ER stress is closely related to autophagy, the interaction between autophagy and ER stress in ischemic stroke is still controversial (Hadley et al., 2018). ER stress may play an important role in neuronal autophagy process under ischemic conditions. Nonetheless, further researches are still needed to confirm this assumption. An increasing number of studies have also shown that the accumulation of reactive oxygen species (ROS) can trigger oxidative stress, leading to autophagy induction in cerebral injury, while targeted inducing or inhibiting autophagy can alleviate or aggravate ROS mediated neuronal damage (Li et al., 2014; Dai et al., 2017; Ahsan et al., 2021; Zhu et al., 2022). According to the inhibitory effect of autophagy on oxidative stress, it could be proposed that targeted manipulation of autophagy process might serve as a promising therapeutic target for ischemic stroke. Collectively, research on strategies for regulating mitophagy or ER stress in cerebral ischemia should be encouraged.

To sum up, from the above analysis, some prominent information can be generalized regarding autophagy in ischemic stroke as follows: 1) Although autophagy plays a key role in ischemic stroke pathophysiology, it is still controversial whether the activation of autophagy is a friend or a foe in the pathogenesis of ischemic stroke. Thus, more in-depth researches are urgently needed. 2) Impaired mitophagy is closely associated with brain damage in ischemic stroke, developing targeted mitophagy methods can serve as a potential strategy for treating ischemic stroke in the future. 3) Autophagy in ischemic stroke-related researches is mainly focused on the field of basic research, with a special focus on genetics and immunity, but research on clinical transformation is still very limited. In the future, more attention should be paid to clinical transformation research. 4) The joint efforts from academic organizations worldwide are in demand to develop novel strategies to therapeutically target autophagy for the identification of new therapeutic agents for the treatment of ischemic stroke. 5) Due to limited research quantity, in the current study, we only analyzed publications from 2011 to 2022, which may not provide a more comprehensive overview of the entire research landscape of autophagy in ischemic stroke. In future research, we will continue to focus on the research trends in this field.

There are several limitations to the present study. Firstly, since only the WOSCC database was employed for literature retrieval in the current study, therefore, some literatures were not included, and citation counts may be also underestimated, which may not truly reflect the current status of all researches in the field of autophagy in ischemic stroke. Secondly, CiteSpace software cannot distinguish between first and corresponding authors, nor can it distinguish between authors with the same name but different units. Last but not the least, because of the limitations in the existing CiteSpace software, there is a lack of unified parameter setting standards (Qin et al., 2022), thus partial data loss will unavoidably occur in software clustering process, which may yield to unexpectedly different analysis results. Therefore, in view of the shortcomings of the existing methods, the design of bibliometric analysis like this study still needs further improvement.

## 5 Conclusion

Based on the above comprehensive bibliometric analysis, it can be seen that research on autophagy in ischemic stroke is still in the ascendant. Our study has uncovered the development of autophagy in ischemic stroke area from 2011 to 2022. By studying previous high-quality articles, this bibliometric analysis provides an objective and quantitative method for evaluating the trends and leading edge of autophagy in ischemic stroke, as well as delivers important insights towards understanding the dynamic evolution over the past decades and the current research hotspots of this field.

## Data Availability

The original contributions presented in the study are included in the article/Supplementary material, further inquiries can be directed to the corresponding authors.
